# Energetic electron assisted synthesis of highly tunable temperature-responsive collagen/elastin gels for cyclic actuation: macroscopic switching and molecular origins

**DOI:** 10.1038/s41598-019-48830-w

**Published:** 2019-08-26

**Authors:** Nils Wilharm, Tony Fischer, Florian Ott, Robert Konieczny, Mareike Zink, Annette G. Beck-Sickinger, Stefan G. Mayr

**Affiliations:** 1 Leibniz-Institut für Oberflächenmodifizierung e.V. (IOM), Permoserstr. 15, 04318 Leipzig, Germany; 2 Biological Physics Division, Department of Physics and Earth Sciences, Leipzig University, Linnéstraße 5, 04103 Leipzig, Germany; 3 Institute of Biochemistry, Department of Life Sciences, Leipzig University, Brüderstraße 34, 04103 Leipzig, Germany; 4 Junior Research Group Biotechnology and Biomedicine, Department of Physics and Earth Sciences, University of Leipzig, Linnéstraße 5, 04103 Leipzig, Germany; 5 Division of Surface Physics, Department of Physics and Earth Sciences, Leipzig University, Linnéstraße 5, 04103 Leipzig, Germany

**Keywords:** Materials science, Soft materials, Self-assembly

## Abstract

Thermoresponsive bio-only gels that yield sufficiently large strokes reversibly and without large hysteresis at a well-defined temperature in the physiological range, promise to be of value in biomedical application. Within the present work we demonstrate that electron beam modification of a blend of natural collagen and elastin gels is a route to achieve this goal, viz. to synthesize a bioresorbable gel with largely reversible volume contractions as large as 90% upon traversing a transition temperature that can be preadjusted between 36 °C and 43 °C by the applied electron dose. Employing circular dichroism and temperature depending confocal laser scanning microscopy measurements, we furthermore unravel the mechanisms underlying this macroscopic behavior on a molecular and network level, respectively and suggest a stringent picture to account for the experimental observations.

## Introduction

As most abundant extracellular matrix protein in vertebrates, including humans, collagen constitutes a perfect starting point for developing biomaterials for a plethora of applications, including wound dressings, osteogenic fillers, antithrombogenic surfaces and bioresorable scaffolds^1–4^. For the past decade a paradigm shift from passive towards smart implants that actively interact with their environment is successively being performed. Adaptive stimuli responsive collagen that combines established benefits and broad acceptance with switchability as novel functionality is a stringent development along these lines; it promises applicability in biomimetic and -resorbable active elements for *in vivo* use, including mechanically stimulating scaffolds for tissue engineering, self-adjusting implants or drug delivery systems^5–8^. Attachment of stimuli responsive elements constitutes one route to implement “smart properties” into collagen, which is conventionally realized by biochemical reactions, but can also be facilitated merely by treatment of hydrated blends with energetic electrons, as we demonstrat in the following for collagen/elastin gels. Primarily based on a water-splitting mechanism, the treatment with energetic electrons constitutes a highly attractive alternative to introduce intra- and intermolecular covalent crosslinks without the need for addition of further, potentially hazardous, reagents^9^.

A temperature change is clearly among the most obvious stimuli that has previously been employed for actuation in collagenous systems in a variety of ways, including the sol-gel transition and its manifestation, the shape memory effect, in electron beam crosslinked gelatin^10^. While certainly useful in a variety of applications, the latter is hampered by its one-way nature that requires mechanical back-transformation. In the present work we demonstrate that electron beam modified blends of natural collagen and elastin gels constitute a milestone towards two-way bio actuation. Furthermore, the electron beam treatment sterilizes the samples^9^.

## Experimental Details

### Preparation of gels

Elastin/collagen gels were prepared by mixing rat tail collagen (Collagen, R, 0.4% solution, Cat. No. 47256.01; SERVA Electrophoresis, Germany) and bovine skin collagen (Collagen G, 0.4% solution, Cat. No., L 7213; Biochrom, Germany) with a ratio of 1:2. The final concentration of 2 mg ml^−1^ was achieved by mixing the collagen solution with a buffer containing Na_2_HPO_4_ (Cat. No. 71636; Sigma-Aldrich Chemie GmbH, Germany) and NaHPO_4_ (Cat. No. 71507; Sigma-Aldrich Chemie GmbH, Germany) to reach a pH value of 7.5 and a total phosphate molarity of 200 mM. Elastin powder (Cat. No. 6527; Elastin, Sigma-Aldrich, Germany) was added to the buffer solution to give an overall concentration of 1 mg ml^−1^ which results in a mass ratio of elastin to collagen of 1:2. The final solution with a volume of 4 ml was cast in cubic molds (2.4 cm × 2.4 cm × 1.6 cm) and placed in an incubator at 37 °C and 100% humidity for 24 h for polymerization. After rinsing the gels twice with distilled water, they were stored in distilled water at room temperature.

### Electron beam treatment

The obtained gels were irradiated with a 10 MeV linear electron accelerator (MB10-30MP; Mevex Corp., Canada). The resulting beam has a pulse duration of 15 µs and a frequency of 180 Hz. The number of pulses finally determines the dose (1 kGy = 1 J kg^−1^) whereas pulse number and dose are calibrated using a graphite dosimeter. The dosimeter heats up during irradiation – the increase of temperature is known so the dose can be read from the temperature increase. 50 kGy to 90 kGy were used with steps of 5 kGy to bypass heat-induced degradation of the samples. Additionally, a fan amplifies cooling of the samples. After treatment the samples were stored tat 37 °C and 100% humidity for 24 h before subjecting them to measurement.

### Setup for characterization of switching behavior

The setup is composed of a home-built heating stage that consists of a 10 cm by 10 cm aluminum plate with a heating foil (Thermo Polyester Heating foil, Cat. No. 532878 – 62, Conrad, Germany) attached below. The temperature of the foil is controlled by an ATMega328 microcontroller, mounted on an Arduino board (Cat. No. 191789 – 62) while the feedback temperature is read from a measuring sensor (Linear IC, Temperature sensor, Cat. No. 1123323 – 62, Conrad, Germany) which is immersed in the measurement container (2.4 cm × 2.4 cm × 1.6 cm, plastic). The gel (sample height: 3 mm) is placed in this container and fully covered with water. The temperature is set to reach 45 °C and then cool down to 30 °C. The heating stage with the gels is filmed with a camera (UI-3240ML-C-HQ; IDS, Germany) at 0.9 frame s^−1^ until the heating protocol is completed. The long-term heating measurement uses a heating program where the temperature varies between 28 °C and 42 °C for twenty cycles while dwelling at each temperature for 5 minutes. Assuming a linear approximation of the heating profile the heating rate was ~0.04 °C sec^−1^. Imaging was conducted as described above.

### Image analysis

All movies were analyzed with a self-written python code. The movies were first split into individual greyscale images; the respective region of interest (ROI) was subsequently cropped and adjusted for brightness. In the series on dose dependence of switching behavior, a circle is fitted to the top view sample area of the gel using a gradient method, while for the temperature cycle experiments a threshold algorithm splits the image into “white” and “black” for the gel sample surface and background, respectively. The number of “white” pixels was employed as a measure for the gel area, assuming homogeneous gel deformation. The pixel to area and frame to time conversions were conducted using a reference object of known size in the image (i.e. ePtri dish) and the data acquisition rate of the camera/temperature sensor, respectively. A Gaussian filter was finally employed for signal smoothing (Python package: scipy.ndimage.filters.gaussian_filter). The filter modifies the input signal by convolution with a Gaussian function with a given standard deviation σ. Generally, higher σ reduces noise more efficiently, but at the same time may lead to loss of shape of the input function. Here, a σ of 50 was chosen since this was necessary for the proper determination of the 1^st^ derivative.

### Refinement of gels for CD measurements

For all doses, gels were placed in 10 ml volume of distilled water in a 50 ml tube each and stirred on a magnetic stirrer (C-MAG HS 7 digital, IKA, Germany) for 24 h and 1500 rpm. The resulting suspension is filtered through a 70 µm cell strainer (Cat. No. 10199-656, VWR International) and a 220 nm syringe filter (Cat. No. 99722, TPP, Switzerland). The obtained solution is transparent and proves to be suitable for the desired measurements.

### Circular dichroism

Circular dichroism was measured using a JASCO Corp., J-715 (JASCO, USA). The solutions obtained as described above were measured in a quartz cuvette with 2 mm path length. Before measurement a quick scan was performed (250 nm to 190 nm, 50 nm min^−1^, wavelength resolution: 0.2 nm) to ensure the HT value remains below 600 V until 195 nm. For every measurement series (250 nm to 190 nm, 20 nm min^−1^, wavelength resolution: 0.2 nm) a water reference was measured which was subtracted from the spectra. Each sample was heated with 1 °C min^−1^ and measured at intervals of 5 °C or 10 °C., respectively with the built-in heating device. Three spectra were recorded for each temperature and the mean value was calculated automatically. In some cases, the spectra were normalized to the norm of each spectrum. Irradiated solutions of elastin and collagen in phosphate buffer (see above) with a concentration of 2 mg ml^−1^ were filtered as described. After diluting them with distilled water until the HT remained below 600 V until 195 nm they were measured as described above. A Gaussian filter was finally employed for signal smoothing (Python package: scipy.ndimage.filters.gaussian_filter). The filter modifies the input signal by convolution with a Gaussian function with a given standard deviation σ. Generally, higher σ reduces noise more efficiently, but at the same time may lead to loss of shape of the input function. Here, a σ of only 10 was chosen since preservation of input curve shape was paramount for correct interpretation.

### Confocal laser scanning microscopy (CLSM) based pore size analysis

A 60 kGy gel was placed in a solution of TAMRA (Cat. No. 90022, Bio trend, Germany) with a concentration of 0.05 mg ml^−1^ for 1 night at room temperature. It was then cut into a disk of 1 cm and placed into a custom-built sample holder, which was mounted in the incubator of a LSM microscope (TCS SP8, Leica, Wetzlar, Germany). The temperature was set to reach 45 °C with 100% humidity. Measurements were conducted with 20x objective, size of one tile: 200 µm², 25 tiles, excitation wavelength: 635 nm. After stitching the tile scans with the Leica software LAS X a video of 737 frames length was created. Finally, a 2D pore size analysis was performed with a self-written python program. This program performs an advanced implementation of 2D bubble analysis where the largest circle fitting in spaces between segmented fibrils is considered a “pore”. The radius of such a circle is considered as the pore size of that respective pore.

## Results

### Heating behavior

Blends of collagen and elastin gels with a ratio of 2:1, which equals physiological composition of lung tissue^11^, were cast in cubic molds of size 2.4 cm × 2.4 cm × 1.6 cm and subsequently irradiated with 10 MeV electrons using doses in the range of 50 and 90 kGy, as detailed in the methods and materials section. The advantages of the use of two types of collagen and their respective concentration was described elsewhere^12^.

Our choice of elastin as a component was motivated by the extended ↔ β-turn transition that is established to occur in elastin with increasing temperature and to reveal an accompanying dimensional change^13^. As starting hypothesis it was envisioned to employ these individual elastin chains as “molecular actuators” to exert forces on the surrounding collagen network, to which they are covalently bonded due to electron irradiation.

**Figure 1:**
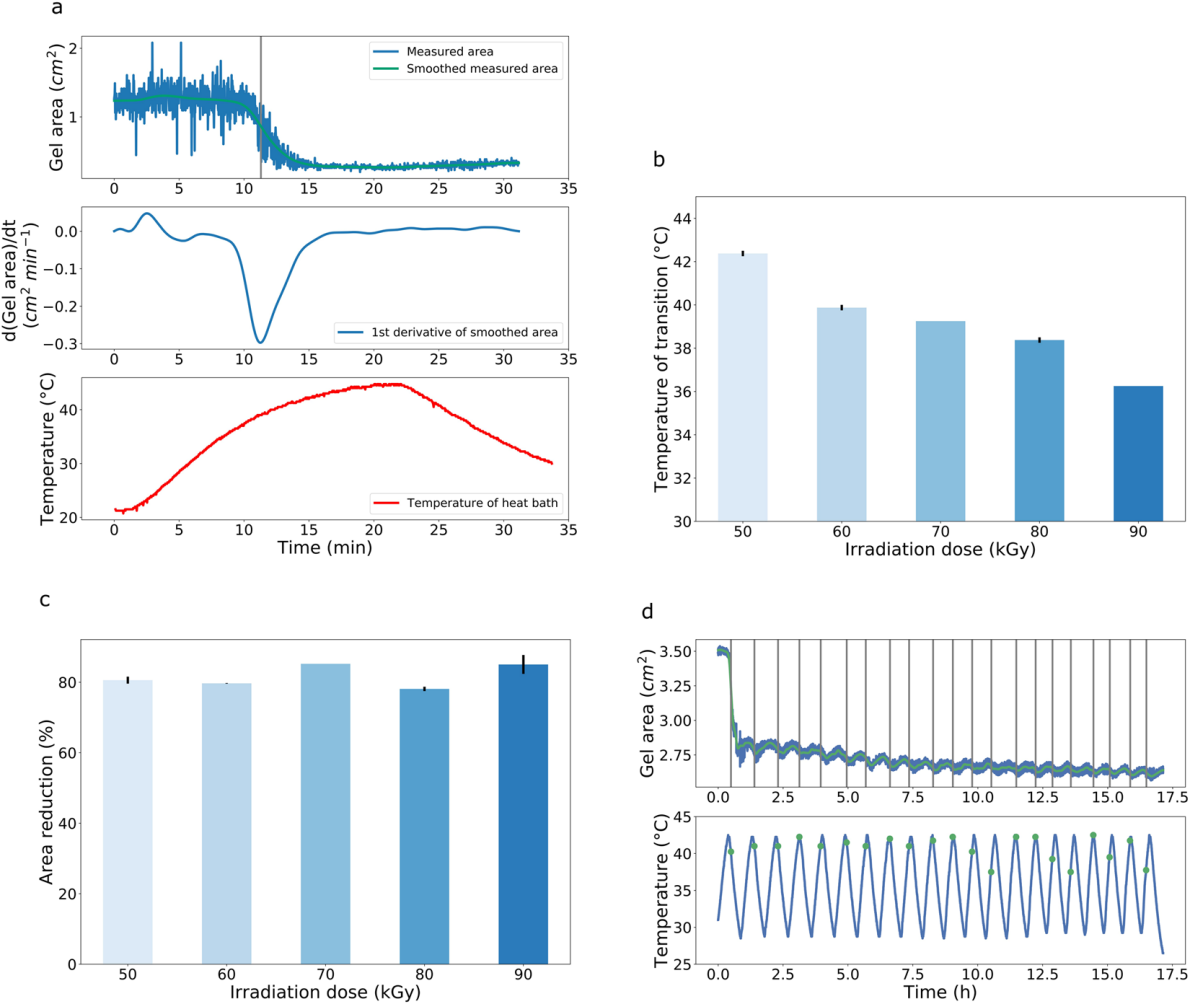
Temperature induced size changes in collagen/elastin gels as function of electron dose. (**a**) Sample area in top view and its derivative when traversing a temperature ramp, exemplarily shown for 60 kGy electron dose. (**b**) The transition temperature as function of dose. (**c**) Relative size change (“stroke”) as function of dose. (**d**) Periodic sample area changes during temperature cycling, exemplarily shown for 60 kGy electron dose; vertical lines denote the inflection points and the dots in the lower plot mark the corresponding temperatures.

As Fig. 1 demonstrates, this envisioned behavior is, in fact, observed: Fig. 1a exemplarily displays the size of our collagen/elastin gel sample after exposure to an electron dose of 60 kGy as function of temperature, as extracted from Video 1 (Supporting Information). The sample area in top view reveals a dramatic decrease of more than 80% when traversing the transition temperature - a clear fingerprint of switching with signatures of a phase transition. It is also apparent that annealing above the transition temperature does not further increase contraction. In fact, contraction slows down even while the temperature still increases which corroborates presence of a sharp transition temperature. This effect becomes clearer at higher doses; e.g. for 90 kGy the transition temperature lies at around 36 °C whereas heating continues until 45 °C but contraction is already completed below 40 °C (not shown). After turning off heating, the gel showed a small recovery phase until the measurement was finished. The recovery rate is much smaller than the contraction rate despite identical cooling and heating rates, respectively. The almost symmetric first derivative of the smoothed signal was used to determine the transition temperature. The dependence of the latter on dose is plotted in Fig. 1b (two samples per dose from the same batch were measured, except for 70 kGy), showing an almost linear decrease of transition temperature from 43 °C to 36 °C for doses increasing from 50 kGy to 90 kGy, respectively. As this temperature interval coincides with the physiological relevant regime this promises tunability in potential biomedical applications. A very interesting observation relates to the stroke during transformation, which does not significantly depend on irradiation dose (Fig. 1c) and is thus decoupled from the dose dependence of transition temperatures (Fig. 1b). Similarly, the contraction speed proved to be independent of dose as well and to be only a function of heating rate within the investigated regime. For instance, we observed by visual inspection that a collagen-elastic gel irradiated with 60 kGy contracts instantaneously when placed in 45 °C hot water, which demonstrates the ability for fast switching. As we will show below, systematic studies reveal a slight dependence of contraction temperature on heating rate, however only within (1–2) °C for heating rates different by more than an order of magnitude. Figure 1d demonstrates the nearly reversible switching behavior during temperature cycling, exemplarily for a 60 kGy collagen/elastin gel; the results are extracted from the original videos included in the Supporting Information. After an abrupt initial decrease, the gel volume almost perfectly follows the temperature, except for an irreversible contribution of 0.34% ± 0.22 that is not recovered within one heating cycle and thus leads to a small, but steady volume decrease. As for the transformation temperature, no shift is detected within the first 20 cycles.

### Pore size analysis

**Figure 2:**
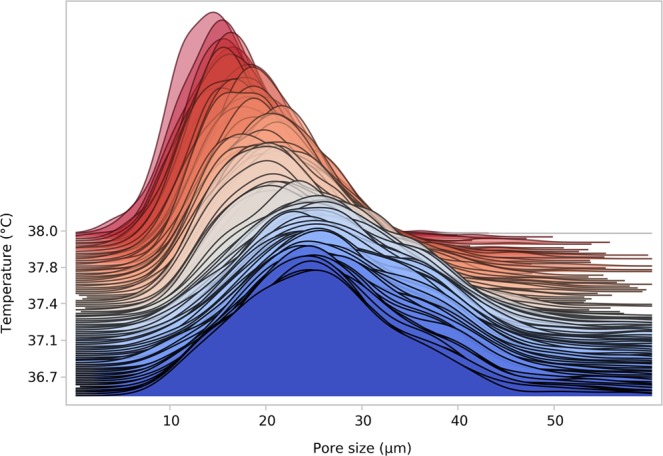
Pore size distribution of a collagen/elastin gel irradiated with 60 kGy during temperature increase. The lines denote calculated histogram densities.

To address the foundations of the macroscopic volume response on the scale of the collagen/elastin network, the 3D network structure was imaged with CLSM (confocal laser scanning microscopy) throughout the transition temperature using a variable temperature stage (the corresponding original video of the stained gel in the CLSM is included in Video 4 - Supporting Information). The gel shows a minor drift since it cannot be fixed within the measurement chamber without disturbing the contraction process. While no significant morphological changes are observed before and after the transition, the gels starts drifting once reaching the transition temperature, presumably because water is squeezed out. Contraction occurs homogeneously within the system without signs of nucleation scenarios. A pore detection algorithm (described in the Supporting Information) is subsequently employed to extract the pore size distribution as function of temperature that shows a shift from larger (*P*_*max*_ ~ 26 µm) to smaller (*P*_*max*_ ~ 15 µm) pore sizes as well as a narrowing of the distribution when traversing the transition (Fig. 2). The pore size distribution ends abruptly due to gel contraction related drifts out of the observable imaging region of the microscope. However, we are confident, that the end of the pore size curve also marks the end of contraction process, as the process was completed by then. The slight increase in pore size from 36.7 °C to 37.1 °C might be an artifact but could also be explained with temperature induced untangling of the fiber structure. However, this remains speculative and will be dealt with in future experiments.

Finally, by combining Figs 1 and 2, the dependence of transition temperature on heating rate can be discerned, as ~0.04 °C sec^−1^ and ~0.0014 °C sec^−1^ (a factor of 30 slower) were employed, yielding 40 °C and 37.5 °C for a 60 kGy gel, respectively.

The observed shift to lower temperatures for lower rates is in line with the general observations in thermally activated transitions. In fact, as elastin is much more susceptible to temperature rate than collagen^14,15^, dominance by elastin is also expected presently. This notion is additionally corroborated by the vanishing contribution of collagen structure itself to volume changes.

### Protein secondary structure

As macroscopic strains and underlying network contractions are induced by changes of the protein secondary structure, circular dichroism (CD) measurements are employed to establish this relation. On the level of the protein structure we have demonstrated that the solutions and the gels indeed respond to a temperature increase over the whole heating range from 25 °C to 45 °C. The secondary structure seems to adapt to the temperature in a gradual way, which implies that restructuring is happening until a certain threshold is reached and the system collapses to the contracted phase.

First, we showed, that the collagen/elastin gel can be described with the individual spectra of elastin and collagen.

**Figure 3:**
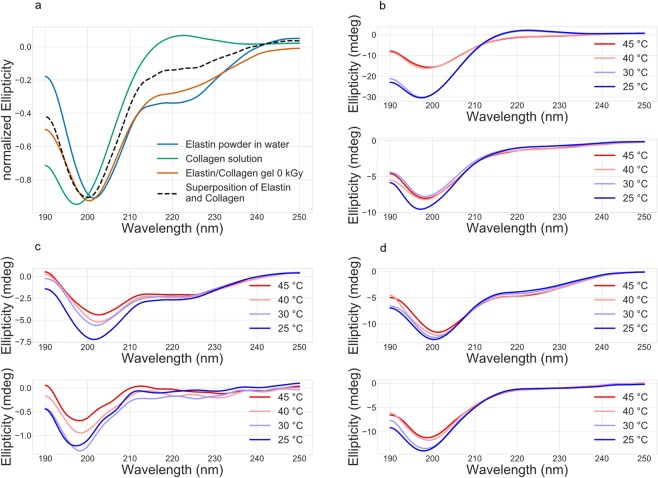
Circular dichroism measurements of solutions of differently irradiated collagen/elastin gels *(All plots: top: 0 kGy*. *bottom: 60 kGy)*. (**a**) Comparison of elastin and collagen solutions. (**b**) Heating behavior of differently irradiated collagen solutions. (**c**) Heating behavior of differently irradiated elastin solutions. (**d**) Heating behavior of differently irradiated collagen/elastin gels after dispersing them in solution. Changes in the curve around 200 nm and 220 nm indicate changes in the protein structure.

Figure 3a represents the curves of untreated samples of elastin, collagen, a collagen/elastin gel and the superposition of the individual elastin and collagen curves. The gel (red curve) can be roughly approximated with the superposition of the elastin curve and the collagen curve (dotted black curve) with the exception of a reduced PPII helix content of collagen (220 nm) in the blend mixture as expected from the superposition.

Next, untreated elastin and collagen solutions are compared to samples, treated with 60 kGy. Figure 3b presents the well-investigated helix → coil transition of collagen^16^. At around 37 °C the helix is destabilized due to thermal motion and forms a random arrangement of structures. The peak at 197 nm represents the coil proportion while the peak at 222 nm shows the triple helix. The bottom plot shows the curve of the 60 kGy irradiated collagen solution and displays that the changes in the structures due to irradiation occur for the 222 nm peak and for the 197 nm peak. Therefore, there is a reduction of the helical and coil content during irradiation. This seems to contradict our findings regarding FTIR measurements on irradiated collagen gels: We showed that the gels exhibit only minor changes in the chemical structure upon irradiation^9^. We propose that the connectivity of amino acids is, if any, slightly altered as opposed to the folding state of the protein chain.

Temperature increase seems to have nearly no effect. The gel is arrested in the room temperature (RT) configuration. This is a first indication that collagen cannot contribute to the contraction process since this would derive from a major change in secondary structure^17^. The CD spectra of the elastin solution corroborate this notion. Figure 3c displays the CD of the solution of elastin at different temperatures. In agreement with its alpha-helical structure a pronounced plateau at 222 nm was observed. In addition, a negative Cotton effect at 203 nm was found indicating some random coil. This was significantly decreased and shifted towards 207 nm with increasing temperatures, indicating a loss of secondary structure. The shift and decrease from 203 nm to 207 nm upon heating has been attributed to an increase in order from random coil to beta turn^13^.

After irradiation of the solution with 60 kGy the secondary structure of the protein changes as indicated by decrease and shift of the negative Cotton effect from 203 nm to 197 nm and the loss of the band at 222 nm. Accordingly, the alpha-helical structure of elastin is lost after irradiation and changes the protein to random coil. This feature of partial protein denaturation by electron beam treatment has been found in the case of egg white protein at 16 kGy^18^. Furthermore, it was shown, that a positive peak between 206 nm and 212 nm is characteristic of type II beta turns, which are one driving factor for contraction^13^. Irradiation hampers the temperature induced formation of type II beta turns. The major change in the curve shape leads to the conclusion, that electron beam treatment of elastin induces more changes than in collagen. The bottom plot in Fig. 3c shows that elastin still responds to temperature changes in the region of 197 nm as opposed to collagen.

We consider this as a direct indication that the elastin biopolymer subsystem governs the contraction process. The results for the broken-down gels (Fig. 3d) finally indicate a shift in the mechanism of contraction from the coil and helix regions to merely the coil region upon electron irradiation. We speculate that the slightly nonlinear behavior of transition temperature as a function of dose (Fig. 1b), particularly the weak plateau around 70 kGy, might be a fingerprint of this behavior. Electron beam treatment successively shifts the peak from 200 nm to 197 nm which coincides with disappearance of the plateau region. This leads to the conclusion that irradiation increases the amount of coil structures and decreases the alpha-helical content as detected before for elastin. The broken-down gel shows only little temperature dependence of the unirradiated gel, but stronger effects after treatment with 60 kGy of the band at 197 nm which is characteristic for the unstructured protein. Taken together, the alpha-helical structure of elastin is strongly reduced by irradiation with 60 kGy, both for elastin itself as well as for the collagen/elastin gel. The same holds true for the triple-helix of collagen whereas the random coil structure contribution is temperature insensitive as well as little affected by irradiation.

## Summary

To wrap things up, our CD measurements indicate a gradual change in elastin structure due to heating, which is in line with a gradual decrease of the radius of gyration reported for elastin-like polymers with increasing temperature in previous studies^19^ and presently results in build-up of an increasing compressive stress on the network backbone. In contrast to well-established tensile loading^20^, the network backbone is susceptible to Euler buckling under compressive loading, leading to network collapse once a critical load is exceeded^21^. We propose that the observed sharp contraction transition occurs exactly at the point, where the elastin-generated stresses equal the critical load σ_crit_ of the network backbone. Thus the network backbone acts as a “mechanical switch” that virtually binarizes the continuous stress σ exerted by the elastin subsystem into two regimes, σ ≤ σ_crit_ and σ > σ_crit_. While Euler buckling *per se* is reversible, failure of individual fibers contributes to irreversible contributions during cycling, as observed in Fig. 1d.

To conclude, we have demonstrated electron beam assisted reagent-free synthesis of temperature responsive collagen/elastin biological gels with largely reversible volume strokes up to 90% and distinct switching at a temperature, which is preadjustable in the physiological relevant range by the applied electron dose. While we have clarified the underlying mechanisms on a molecular and network level, our “bio-only” reversible actuator opens up exciting new possibilities in biomedicine.

## Supplementary information

Video 1

Video 2

Video 3

Video 4

SUPPLEMENTARY INFO
