# Supplementary material for: Energetic electron assisted synthesis of highly tunable temperature-responsive collagen/elastin gels for cyclic actuation: macroscopic switching and molecular origins
Source: Sci Rep. 2019 Aug 26;9:12363. doi: 10.1038/s41598-019-48830-w (PMC6710254; doi:10.1038/s41598-019-48830-w)

**Energetic electron assisted synthesis of highly tunable temperature-responsive collagen/elastin gels for cyclic actuation: macroscopic switching and molecular origins – Supporting Information**

*Nils Wilharm, Tony Fischer, Florian Ott, Robert Konieczny, Mareike Zink, Annette G. Beck-Sickinger, and Stefan G. Mayr*

M. Sc. Nils Wilharm, Robert Konieczny, Prof. Dr. Stefan G. Mayr

Leibniz-Institut für Oberflächenmodifizierung e.V. (IOM)

Permoserstr. 15

04318 Leipzig, Germany

nils.wilharm@iom-leipzig.de

stefan.mayr@iom-leipzig.de

M. Sc. Tony Fischer

Biological Physics Division,

Department of Physics and Earth Sciences,

University of Leipzig

Linnéstraße 5

04103 Leipzig, Germany

M. Sc. Florian Ott, Prof. Dr. Annette G. Beck-Sickinger

Institute of Biochemistry,

Department of Life Sciences,

University of Leipzig

Brüderstraße 34

04103 Leipzig, Germany

M. Sc. Nils Wilharm, Prof. Dr. Mareike Zink

Junior Research Group Biotechnology and Biomedicine,

Department of Physics and Earth Sciences

University of Leipzig

Linnéstraße 5

04103 Leipzig, Germany

M. Sc. Nils Wilharm, Prof. Dr. Stefan G. Mayr

Division of Surface Physics,

Department of Physics and Earth Sciences,

University of Leipzig

Linnéstraße 5

04103 Leipzig, Germany

**Experimental movies / videos**

Video 1: Size change upon temperature increase from 295 K to 318 K in a collagen/elastin gel, treated with an electron dose of 60 kGy, immersed in water. The circle denotes the determination of the gel circumference by the evaluation algorithm.

Video 2: Reversible size change during temperature cycling 295 K to 318 K in the sample shown in Video 1. The movie covers, in fast motion, the experimental observations of 16 h after adjusting brightness and contrast for each frame. A syringe pump continuously supplied water to the incubator chamber to ensure coverage of the sample with water throughout the experiment.

Video 3: Binarized and faster motion version of Video 2. The video shows an artifact in the bottom right corner, where light reflected by the water surface lies within the threshold regimes of the gel detection algorithm. However, due to its systematic nature, this effect merely results in an offset in the absolute sample area throughout the experiment.

Video 4: Original video of a CLSM measurement on structural changes in the collagen / elastin network, irradiated with an electron dose of 60 kGy, upon temperature increase from 300.6 K to 311.4 K.

**Supplementary figures**

Figure S1: Same data as Figure 3d-bottom but with higher temperature resolution. Some temperatures in Figure 3d have been omitted for better readability.


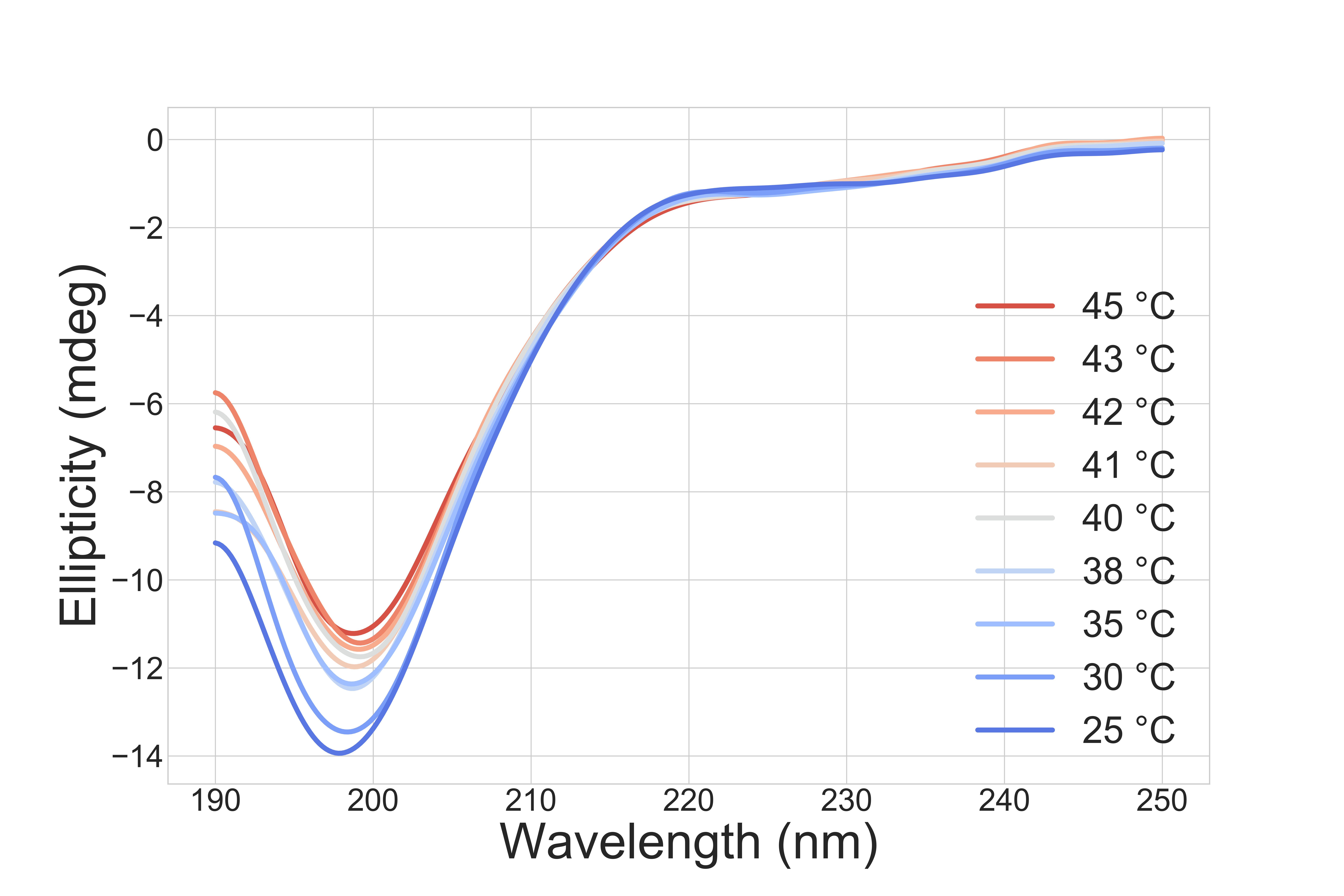


Figure S2: Ellipticity evolution of the 197 nm peak in Figure 4. A 4 parameter logistic (4PL) fit was employed since proteins usually show this kind of saturation behavior under temperature increase.


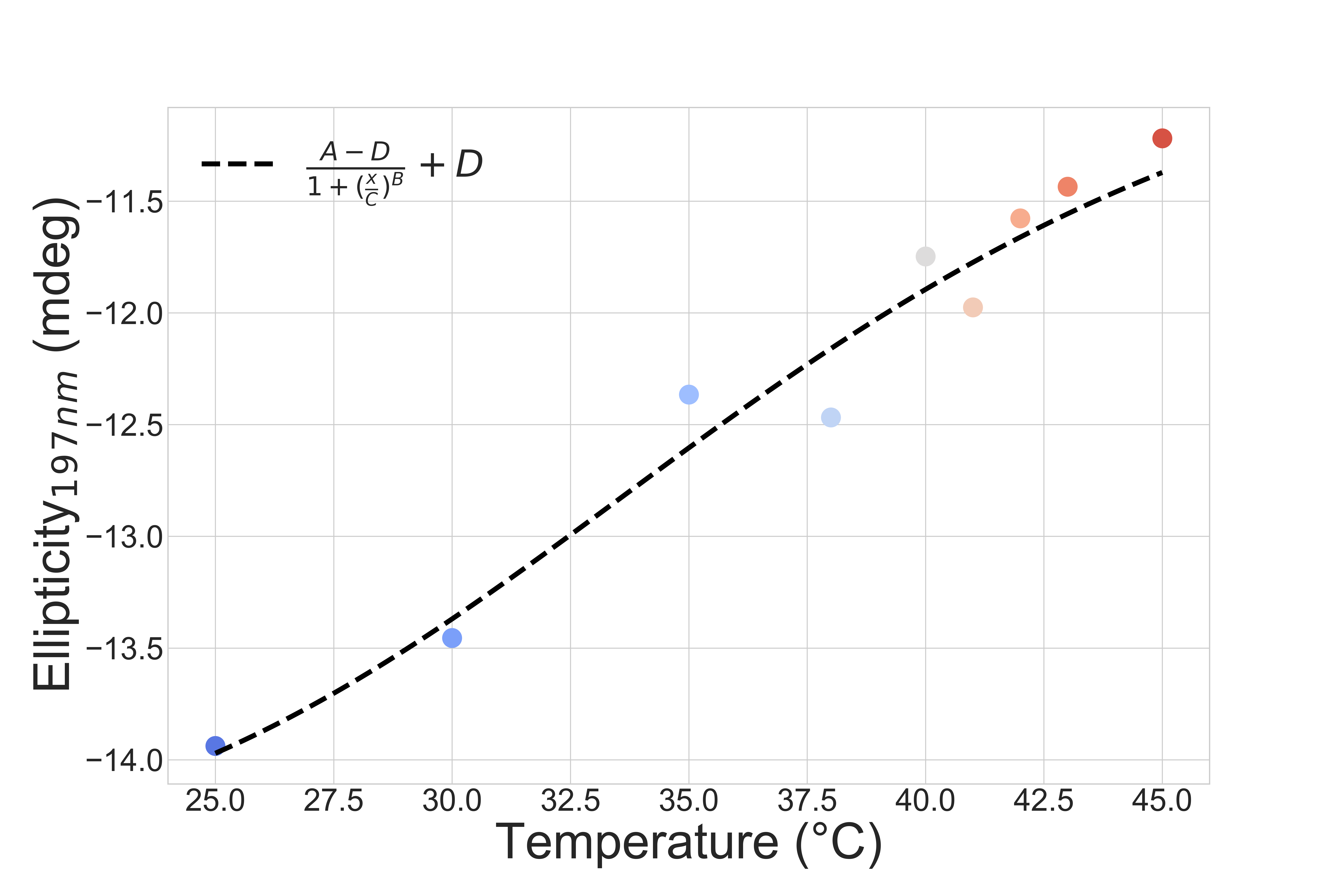

Supplement: Supplementary file 5 — SUPPLEMENTARY INFO [file 41598_2019_48830_MOESM5_ESM.doc]
